# Immunization of Mice with Recombinant Mosquito Salivary Protein D7 Enhances Mortality from Subsequent West Nile Virus Infection via Mosquito Bite

**DOI:** 10.1371/journal.pntd.0001935

**Published:** 2012-12-06

**Authors:** Krystle L. Reagan, Carlos Machain-Williams, Tian Wang, Carol D. Blair

**Affiliations:** 1 Arthropod-borne and Infectious Diseases Laboratory, Department of Microbiology Immunology and Pathology, Colorado State University, Fort Collins, Colorado, United States of America; 2 Department of Microbiology and Immunology, Department of Pathology, University of Texas Medical Branch, Galveston, Texas, United States of America; National Institute of Allergy and Infectious Diseases, United States of America

## Abstract

**Background:**

Mosquito salivary proteins (MSPs) modulate the host immune response, leading to enhancement of arboviral infections. Identification of proteins in saliva responsible for immunomodulation and counteracting their effects on host immune response is a potential strategy to protect against arboviral disease. We selected a member of the D7 protein family, which are among the most abundant and immunogenic in mosquito saliva, as a vaccine candidate with the aim of neutralizing effects on the mammalian immune response normally elicited by mosquito saliva components during arbovirus transmission.

**Methodology/Principal Findings:**

We identified D7 salivary proteins of *Culex tarsalis*, a West Nile virus (WNV) vector in North America, and expressed 36 kDa recombinant D7 (rD7) protein for use as a vaccine. Vaccinated mice exhibited enhanced interferon-γ and decreased interleukin-10 expression after uninfected mosquito bite; however, we found unexpectedly that rD7 vaccination resulted in enhanced pathogenesis from mosquito-transmitted WNV infection. Passive transfer of vaccinated mice sera to naïve mice also resulted in increased mortality rates from subsequent mosquito-transmitted WNV infection, implicating the humoral immune response to the vaccine in enhancement of viral pathogenesis. Vaccinated mice showed decreases in interferon-γ and increases in splenocytes producing the regulatory cytokine IL-10 after WNV infection by mosquito bite.

**Conclusions/Significance:**

Vector saliva vaccines have successfully protected against other blood-feeding arthropod-transmitted diseases. Nevertheless, the rD7 salivary protein vaccine was not a good candidate for protection against WNV disease since immunized mice infected *via* an infected mosquito bite exhibited enhanced mortality. Selection of salivary protein vaccines on the bases of abundance and immunogenicity does not predict efficacy.

## Author Summary

West Nile virus (WNV) is a mosquito-borne flavivirus found on all continents except Antarctica. Humans and equines are not part of the natural transmission cycle, but when they become infected severe illness or death can result. There is no human vaccine for WNV available, so novel approaches to preventing infection are needed. Mosquito saliva deposited with WNV alters the immune response of the bitten host and potentiates virus transmission and pathogenesis. Previous research with pre-exposure to arthropod salivary proteins showed promising results in blocking the transmission of malaria and *Leishmania* parasites, thus we hypothesized a similar outcome for vaccination with a MSP in protection from arbovirus disease. Unexpectedly, our results showed that administration of a vaccine consisting of a recombinant mosquito salivary protein (rD7) and subsequent mosquito transmission of WNV led to more severe disease and increased death rates in mice. Additionally, when serum from vaccinated mice was transferred to naïve mice, those animals also succumbed to severe mosquito-transmitted WNV disease, suggesting that anti-rD7 antibodies elicited by the vaccine played a role in enhanced disease. We conclude that the rD7 protein vaccine we developed is not a suitable candidate for altering the host immune response to WNV infection to provide increased protection from disease.

## Introduction

With its emergence in the Western Hemisphere in 1999, WNV has become a widespread human and veterinary medical concern in North America [1], [2] along with other temperate and tropical regions of the world. WNV is a positive-sense RNA flavivirus and a member of the Japanese encephalitis virus serogroup. The virus is maintained in a transmission cycle between birds and mosquitoes, primarily of the genus *Culex*. In North America, *Cx. tarsalis, Cx. quinquefasciatus,* and *Cx. pipiens* are reported to be the primary vectors for WNV [3]. Infection of tangential hosts such as humans and equids can result in a spectrum of outcomes ranging from asymptomatic to febrile to severe neurologic disease including meningitis, encephalitis and death. Although successful equine vaccines have been developed and are widely available, no human vaccines are currently available.

Upon natural transmission, arthropod-borne pathogens enter the host together with a complex array of vector salivary proteins [4]. The effects of and immune responses to these proteins have been areas of active vector biological research. Arthropod saliva contains both anti-hemostatic and immunomodulatory factors. Vasodilatory factors, anticoagulants and inhibitors of activation of the plasma contact system [4], [5] aid the arthropod in obtaining a blood meal. Immunomodulation by saliva creates an environment in the vertebrate host that is favorable for enhanced infection by some pathogens, including both parasites and viruses [6], [7], [8], [9], [10], [11]. Mosquito saliva has been shown to induce increases in levels of Th2-type cytokines and decreases in Th1-type cytokines [12], which are not favorable for an effective immune response against infection by viruses such as WNV [13], [14], [15], [16]. Hypothetically, a mosquito salivary protein (MSP) vaccine that could favorably alter saliva-induced immunomodulation would be protective against WNV infection delivered by a mosquito bite. MSPs are highly immunogenic and exposure elicits antibody development in humans and other animals [17], [18]. In studies with leishmaniasis-transmitting sand flies [19] and malaria-transmitting anopheline mosquitoes [20], pre-exposure to salivary proteins was shown to decrease the pathogenesis of the transmitted parasites, although the results with mosquito saliva exposure were not replicated in more recently published work [21]. In addition, pre-exposure to the bites of *Aedes aegypti* led to an enhancement of *Ae. aegypti*-transmitted WNV infection [17].

In our previous studies, mice vaccinated with salivary gland extract from *Cx. tarsalis* in the presence of an adjuvant and subsequently challenged with WNV had decreased viral titers in the brain at four days post-infection and increased levels of IFNγ, TNFα, and IL-10 (Machain-Williams *et al.*, submitted). These results suggest that development and administration of a MSP vaccine could potentially shift the Th1–Th2 polarization normally induced by mosquito saliva and induce protection from mosquito-transmitted WNV disease.

The D7 family proteins are conserved across multiple arthropod species and specifically expressed in the salivary glands of adult female diptera. This family of proteins is highly immunogenic and found at high concentrations in mosquito saliva, suggesting they are good candidates for a vaccine that favorably alters the host immune response to mosquito-transmitted arboviruses [22]. In this study, we expressed recombinant D7 (rD7) protein in cultured mosquito cells and tested its effect as a vaccine in mice subsequently fed upon by WNV-infected *Cx. tarsalis* mosquitoes.

## Materials and Methods

### Cells and Virus

C6/36 (*Aedes albopictus*) cells were cultured in Leibovitz-15 medium (Gibco, LifeTechnologies; bacterial endotoxin <0.03 EU/ml) containing 10% fetal bovine serum, 100 U/ml penicillin and 100 µg/ml streptomycin, and incubated at 27°C. Vero (African green monkey kidney) cells were cultured in minimum essential medium (MEM) with 10% fetal bovine serum, 100 U/ml penicillin and 100 µg/ml streptomycin. They were incubated at 37°C with 5% CO_2_. WNV- NY99 stocks were produced by infection of Vero cells and titrated by plaque assay.

### Quantitative RT-PCR for WNV RNA

To quantify RNA, qRT-PCR reactions were conducted using Quantitect SYBR Green RT-PCR kit (Qiagen) and a Bio-Rad iCycler iQ5 System (Bio-Rad). Primers were designed to amplify a 70 bp portion of the WNV envelope gene [23] (WNV Env Forward is 5′-TCAGCGATCTCTCCACCAAAG-3′, WNV Env Reverse is 5′-GGGTCAGCACGTTTGTCATTG-3′). Standards used to calculate viral genome copy numbers were PCR amplicons cloned into pCR 2.1 (Invitrogen). Experimental samples and standards were amplified as follows: 50°C for 30 min, 95°C for 15 min, then 40 cycles of 94°C for 15 sec, 60°C for 30 sec, 72°C for 30 sec, 77.5°C for 8 sec.

### Animals

*Cx. tarsalis* (Bakersfield, California) mosquitoes were raised in the insectaries at the Arthropod-borne and Infectious Diseases Laboratory (AIDL), Colorado State University, at 25°C and 80% humidity on a 16∶8 hr light∶dark cycle. Adult mosquitoes were given water and raisins *ad libitum* and offered restrained mice once per week for blood meals and egg production. Mice used for WNV challenge were 6–8 week old female C57/BL 6 strain, purchased from Jackson Laboratories (Bar Harbor, Maine). This study was performed in strict accordance with the recommendations in the Guide for the Care and Use of Laboratory Animals of the National Institutes of Health. The protocol was approved by the Colorado State University Institutional Animal Care and Use Committee, Protocol 08-067A.

### Preparation and characterization of *Cx. tarsalis* salivary proteins

MSP were concentrated and fractionated as previously described [24]. Saliva was collected from 5–7 day-old adult female *Cx. tarsalis* mosquitoes by placement of each proboscis into a 10 µl glass capillary tube filled with immersion oil. Oil and saliva from capillary tubes were mixed with MEM, centrifuged, and the aqueous layer was combined with an equal volume of 20% trichloroacetic acid (TCA) and incubated on ice for 30 min. Precipitated proteins were sedimented by centrifugation and the protein pellet was resuspended in PBS with protease inhibitors (Roche). NuPAGE Bis-Tris 10% polyacrylamide gels (Invitrogen) were used to fractionate salivary proteins. Two hundred fifty ng of MSP were loaded per well using a non-denaturing loading buffer (NuPAGE® LDS sample buffer, Invitrogen). Gels were stained with either Silver Stain Plus (BioRad) or Coomassie blue R-250. After fractionation, proteins were transferred to nitrocellulose membranes. For immunodetection, mouse serum was diluted 1∶20–1∶100 in blocking buffer. Horseradish peroxidase (HRP)-conjugated secondary antibodies were diluted 1∶1000 in blocking buffer. To obtain sufficient quantities to identify the major MSP detected in immunoblots, female mosquito thoraxes were dissected, placed in lysis buffer containing 1% sodium deoxycholate, 1% Triton-X-100, 0.1% SDS and 1 mM PMSF, and mechanically disrupted.

Mass spectrometric analyses were performed by the Proteomics and Metabolomics Facility at Colorado State University. Proteins were concentrated by TCA precipitation and fractionated by PAGE as described above. Individual protein bands were excised and digested in the gel with trypsin. Analysis was performed with an UltraFlex-TOF/TOF mass spectrometer (Bruker Daltonics, Billerica, MA) in positive ion, reflector mode with a 25 kV acceleration voltage, and data were processed using the SNAP algorithm in the FlexAnalysis software (version 2.4, Bruker Daltonics). The combined MS and MS/MS spectra for each sample were searched using the Mascot (version 2.1) database search engine against the NCBInr database using a taxonomy filter for mosquito.

The amino terminal amino acid sequence of the 37 kDa salivary protein was determined by Edman degradation and N-terminal sequencing on an Applied Biosystems Procise Sequencer at the Proteomics and Metabolomics Facility.

### Cloning and sequencing of D7 protein cDNA

RNA was extracted from adult female mosquito thoraxes that were placed in Trizol (Invitrogen) after dissection. cDNA was reverse transcribed from RNA from 5 adult female *Cx. tarsalis* thoraxes using a primer with a GC rich linker region and a poly T tail. A degenerate gene-specific primer was designed for PCR using the N-terminal amino acid sequence as determined above. PCR products were sequenced and a consensus sequence was determined. The 5′-end, which codes for a putative signal sequence, was determined using the GeneRacer 5′RACE kit (Invitrogen). The cDNA sequence has been submitted to the NCBI database.

### Recombinant protein expression

The full length *Cx. tarsalis* D7 cDNA with the native secretory signal sequence and encoding a carboxy-terminal six-histidine tag was inserted into the Sindbis virus infectious cDNA clone pTE 3′/2J [25] for expression from the duplicated 3′ sub-genomic promoter and termed pTE3′CtD7 His. To produce protein, 20 µg of pTE3′CtD7 His, pTE3′/2J (empty vector), or Sindbis virus infectious cDNA clone expressing GFP (pTE5′GFP; control) [26], were linearized using XhoI, and infectious RNA was transcribed *in vitro* and electroporated into cultured C6/36 *Aedes albopictus* mosquito cells. rD7 protein or mock vaccine were purified from pooled medium from pTE3′CtD7 or pTE5′GFP transcript-transfected C6/36 cells, respectively, and recombinant virus-infected cells, using binding of the six-histidine tag to ProBond resin (Invitrogen) under native purification conditions. Vaccine protein concentration determined using BCA protein quantification kit (Pierce) was 100–200 µg/ml.

### Mouse Immunization

Mice were immunized with rD7 protein in complete Freund's adjuvant (CFA; Imject, Pierce) for the primary inoculation, followed by boosters at 2 and 4 weeks with incomplete Freund's adjuvant (Sigma). For each inoculation, 10 µg of rD7 was diluted in sterile PBS to a volume of 100 µl and mixed with an equal volume of adjuvant. Mice were anesthetized and inoculated subcutaneously with 50 µl per site on each quadrant of the back. Mock-immunized mice were inoculated with an equal volume of material purified under identical conditions from the medium of cells transfected with SINV TE5′GFP combined with an equal volume of adjuvant.

### Passive D7 immunization of mice

Serum was collected from mice that had been vaccinated with rD7 at 10 days post final booster immunization. Serum from five animals was pooled and recipient mice were given an intraperitoneal (IP) inoculation of 200 µl of pooled serum from rD7-vaccinated mice, mice that had been exposed to the bites of approximately 25 uninfected *Cx. tarsalis* to promote oogenesis at two-week intervals (∼25 times) over a one-year period, or non-vaccinated, non-mosquito-exposed mice.

### Measurement of D7 antibody by indirect ELISA

Immulon (Thermo Scientific) plates were coated with 50 ng rD7 per well. Serum samples were added in four-fold dilutions from 1∶20 to 1∶5120. The threshold for a positive reading was two standard deviations above the mean of negative control samples.

### WNV infection of mice by mosquito transmission

Five-day-old female *Cx. tarsalis* were given an artificial blood meal containing 1×10^7^ PFU/ml WNV-NY99 in defibrinated sheep blood (Colorado Serum Company) maintained at 37°C in a Hemotek feeder (Discovery Workshops). Blood-fed mosquitoes were separated under cold anesthesia and held for ten days with sugar and water *ad libitum*. At ten days post WNV infection, 15 mosquitoes were placed into each one-gallon carton with one anesthetized mouse. When at least one of the mosquitoes had completed a blood meal, the mouse was removed from the carton. Blood-fed female mosquitoes were tested for WNV RNA by RT-PCR. Mice were monitored twice daily for development of clinical signs and euthanized when signs of severe illness were noted.

### Cytokine measurement

At indicated time points, mouse sera were collected and spleens were removed and dispersed to single cell suspensions. Splenocytes in RPMI medium were incubated with Golgi Plug/PMA/ianomyocin cocktail (BD Pharminogen) at 37°C for 4 hr. The cells were washed, resuspended in RPMI medium, and placed in 96-well round bottom plates. For intracellular cytokine staining, cells were stimulated with either rD7 or a MHC Class I or MHC Class II WNV-specific peptide, incubated with surface-staining markers CD4-FITC and CD8-APC, washed, and fixed with Fix/Perm buffer. Cells from each spleen sample were divided and each aliquot was stained with a PE-labeled antibody to one of the following cytokines: IL-10, IL-4, IFNγ, TNFα, or IL-12. The stained cells were resuspended and read on a CyAn (Dako) flow cytometer. Data were analyzed using Summit software.

To assay cytokine production after *ex vivo* antigenic stimulation, splenocyte medium or sera collected at the same time points were analyzed for MCP-1, IFN-γ, TNF-α, IL-10, IL-6 and IL-12p-70 by cytometric bead array (CBA) assay with a mouse inflammatory cytokine kit (BD Biosciences) using a FACSArray analyzer (BD Biosciences).

### Histopathology

One to five uninfected *Cx. tarsalis* were allowed to take blood meals from the ears of mice that had been rD7-vaccinated, mock-vaccinated, or untreated. At two days post mosquito bite, mouse ears were removed and fixed in formalin, sectioned, stained with hematoxylin and eosin and histologically examined at the bite site. Tissue sections were characterized by a veterinary pathologist for absence or presence and severity of inflammation (grade 1 = minimal to grade 4 = moderately severe) and types of cellular infiltrates.

## Results

### Identification and N-terminal sequencing of *Cx. tarsalis* D7 salivary protein

**Figure 1:**
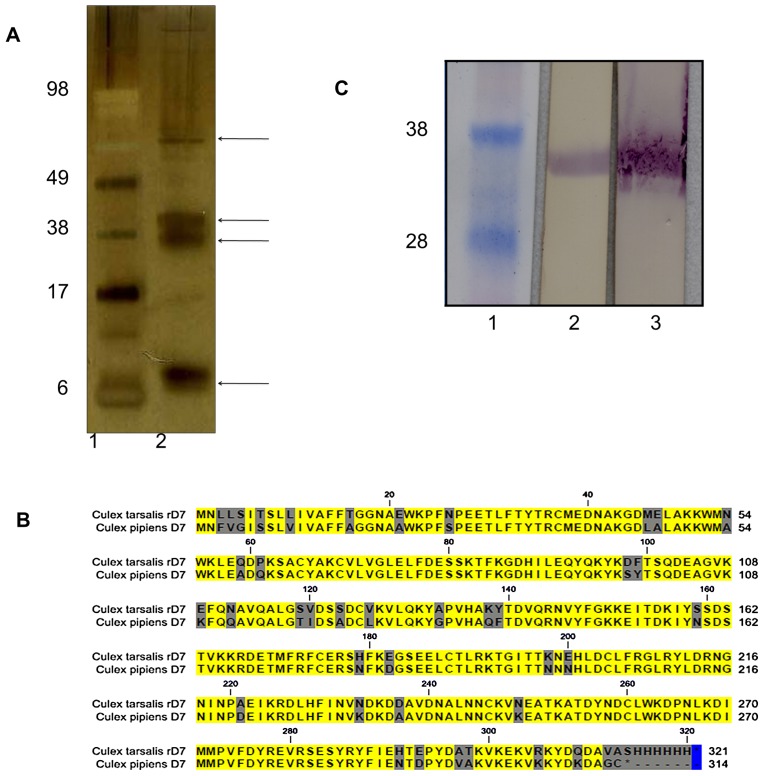
Characterization of *Cx. tarsalis* D7 salivary protein. A. Saliva from adult female *Cx. tarsalis* mosquitoes was collected, proteins were concentrated, PAGE-fractionated and visualized with silver stain (lane 2). Four major proteins, indicated by arrows at right, were present in the saliva with approximate molecular weights of 14 kDa, 36 kDa, 40 kDa, and 60 kDa. Sizes of protein molecular markers (lane 1) are shown at left. B. Amino acid sequence alignment of *Cx. tarsalis* and *Cx. pipiens* D7 proteins. The *Cx. tarsalis* sequence includes the six-histidine tag used for identification and purification of recombinant protein. The amino acid sequences are 85% identical; non-identical residues are shaded. C. Immunoblot using his-tag-HRP antibody or serum from *Cx. tarsalis*-exposed mice to show antibodies made to native protein also bind rD7. Lane 1: Protein size markers. Lane 2: rD7 detected with his-tag HRP antibody. Lane 3: rD7 detected with mouse serum.

To identify D7 proteins of *Cx. tarsalis*, we collected saliva from adult female mosquitoes, concentrated the proteins and fractionated by PAGE. Following silver staining of the gel, four major salivary proteins with molecular weights approximately 14 kDa, 36 kDa, 40 kDa, and 63 kDa were observed (**Figure 1A**). These four proteins were subjected to mass spectrophotometric (matrix-assisted laser desorption/ionization and time of flight [MALDI-TOF]) analysis. The resulting mass fingerprints were searched against the NCBInr mosquito database using Mascot as previously described [24]. Database search results showed that both the 36 and 40 kDa proteins have significant similarity (p<0.05) to two D7 salivary proteins from *Cx. pipiens* (accession numbers 16225986 and 16225983). The 14 kDa and 60 kDa proteins were shown to have high amino acid sequence similarity to a RNA helicase of *Aedes aegypti* and a putative protein from *Anopheles gambiae,* respectively.

To further characterize the 36 kDa D7 salivary protein of *Cx. tarsalis* we performed N-terminal amino acid sequencing by Edman degradation. The 11 residues at the N-terminus were identified as EWKPFNPEETL. Among these 11 amino acids, 9 were identical to those of a *Cx. pipiens* D7 protein.

### Cloning of *Cx. tarsalis* D7 cDNA and expression of rD7 protein

To obtain the nucleotide sequence of the *Cx. tarsalis* 36 kDa D7 cDNA, we used degenerate primers designed from the N-terminal amino acid sequence to conduct 3′ RACE. The N-terminal secretory signal-encoding sequence (60 bp) was obtained by 5′ RACE, resulting in a cDNA of ∼900 bp. Alignment of the derived *Cx. tarsalis* amino acid sequence with that of *Cx. pipiens* D7 protein showed 85% identity (**Figure 1B**).

Recombinant D7 (rD7) protein was expressed by insertion of D7 cDNA under control of the 3′ duplicated subgenomic promoter of a Sindbis virus transducing vector pTE3′2J [25]. High levels of rD7 were secreted into the medium of C6/36 cells infected with SINV TE3′CtD7 His. Histidine-tagged rD7 was purified from pooled medium and its identity verified by mass spectrometric analysis (data not shown). Immunoblot analysis using 6-His-tag antibody or pooled serum from mice that were repeatedly exposed to the bites of *Cx. tarsalis* showed a 37 kDa protein, suggesting that rD7 retained the linear epitopes of the natural D7 protein (**Figure 1C**).

### Induction of IgG1 antibodies, altered cytokine production, and inflammatory cell infiltration in rD7-immunized mice by mosquito feeding

**Figure 2:**
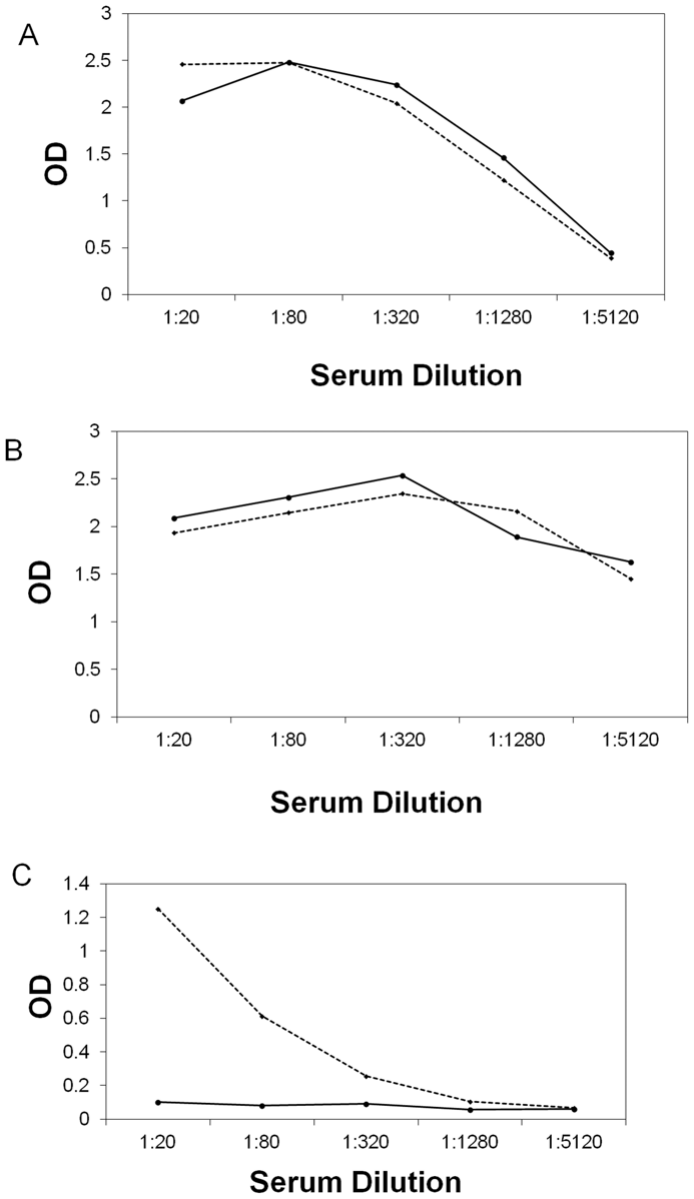
Humoral immune response in rD7-vaccinated mice. Measurement of serum anti-rD7 IgG by ELISA. A. Total IgG specific for rD7. B. IgG1 subtype rD7 antibodies. C.IgG2a subtype rD7 antibodies. Dashed lines: pooled sera of mice repeatedly exposed to the bites of *Cx. tarsalis* (n = 2). Solid lines: sera of mice vaccinated with rD7 (n = 5).

Mice were vaccinated with rD7 protein, mock-vaccinated or exposed to approximately 25 *Cx. tarsalis* mosquito bites every 2 weeks over the course of a year. At 10 days post-final immunization, sera were collected from immunized and naturally-exposed mice and tested for IgG titer to D7 protein by ELISA. Sera from both the rD7-vaccinated mice and mice repeatedly exposed to mosquito bites had IgG titers of 1280 to D7 protein (**Figure 2A**). Mock-vaccinated mice did not produce detectable levels of anti-D7 IgG (data not shown). To characterize the IgG subtypes produced by immunized mice, we conducted ELISAs to detect D7-specific IgG1 and IgG2a antibodies. The rD7-vaccinated mice produced an IgG1 titer greater than 5120 against D7 protein, which was equivalent to that of mice repeatedly exposed to *Cx. tarsalis* mosquito bites (**Figure 2B**). In contrast, immunized mice did not produce anti-D7 IgG2a (**Figure 2C**), whereas the repeatedly exposed mice had IgG2a titers of 320 (**Figure 2C**). These results suggest that immunization with rD7 protein does not induce an identical humoral response to that elicited by exposure to native *Cx. tarsalis* salivary proteins.

**Figure 3:**
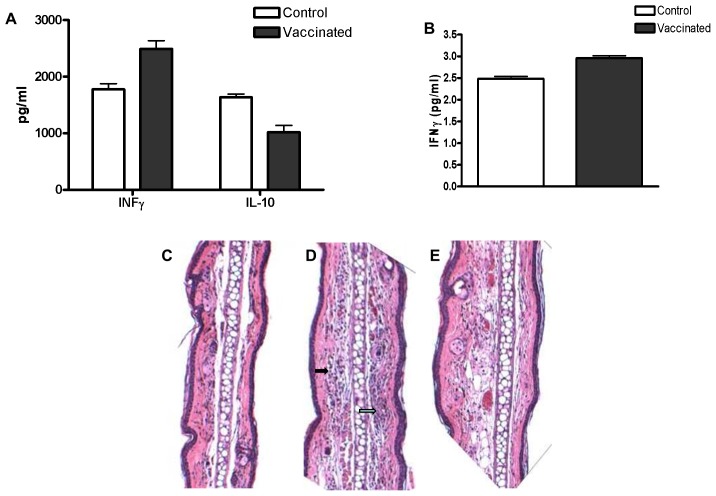
Cellular immune response in rD7-vaccinated mice. A. Assay by CBA demonstrated that at two days post-exposure to the bite of a non-infected *Cx. tarsalis,* splenocytes of rD7-vaccinated mice produced increased IFNγ (p = 0.056) and significantly lower concentrations of IL-10 as compared to mock-vaccinated mice (control) (p<0.05) upon *ex vivo* rD7 stimulation. B. At four days post non-infected *Cx. tarsalis* bite, a significant increase in serum IFNγ was seen in rD7-vaccinated mice compared to mock-vaccinated mice (p<0.05). C–E. Hematoxylin and eosin stained sections of mouse ears at mosquito bite sites at 100× magnification. Mice that were: (C) not vaccinated showed minimal edema and no significant pathological findings, inflammation severity score (mean ± SD) = 0.66±1.15 (n = 3); (D) rD7-vaccinated showed inflammation characterized by edema with prominent neutrophil and mononuclear cell infiltration (arrows), inflammation severity score = 2±1.00 (n = 3); (E): mock-vaccinated, inflammation with edema and little cellular infiltration, inflammation severity score = 0.66±1.15 (n = 3).

To study cytokine production, we exposed immunized mice to the bite of at least one uninfected *Cx. tarsalis* 10 days after the final immunization. Serum and rD7-stimulated splenocyte culture medium were assayed by CBA for MCP-1, IFN-γ, TNF-α, IL-10, IL-6 and IL-12p-70. We found that at day 2 post-feeding, splenocytes of rD7-immunized mice produced more IFN-γ (p = 0.056) and significantly lower levels of IL-10 (p<0.05) than mock-immunized mice following *ex vivo* stimulation with rD7 protein (**Figure 3A**). We also noted a significant increase (p<0.05) in serum IFN-γ levels in rD7-vaccinated mice compared to mock-vaccinated mice at day 4 post-mosquito feeding (**Figure 3B**). Concentrations of other cytokines in culture media and sera, including MCP-1, TNF-α, IL-6 and IL-12, were not significantly different in rD7-immunized mice as compared to mock-immunized mice.

Histopathologic analysis of sub-epithelial connective tissues of ear sections from rD7 protein-immunized mice at day 3 post-uninfected mosquito bite revealed more infiltration of polymorphonuclear leukocytes and mononuclear cells in rD7-immunized mice (**Figure 3D**) than in mock-immunized mice (**Figure 3E**) or untreated mice (**Figure 3C**). Edema at the bite site was noted in non-immunized as well as rD7- and mock-immunized mice, thus inflammation resulting in edema was not specific to rD7 immunization and appears to be a reaction to the mosquito bite. Mean inflammation severity grades for each treatment group were: rD7- vaccinated+mosquito bite = 2.0±1.00; mock-vaccinated+mosquito bite = 0.66±1.15; not vaccinated+mosquito bite = 0.66±1.15.

Differences we observed in responses to mosquito bites between mice that were rD7-vaccinated and those that were either mock-vaccinated or untreated are likely to reflect differences between primary *vs.* secondary immune responses to MSP. Overall, these results suggested that rD7 protein immunization results in higher Th1-type cytokine (IFN-γ) production and more inflammatory cell infiltration at the bite site upon non-infected mosquito feeding.

### Enhanced WNV disease in rD7-vaccinated mice after WNV infection by mosquito bite

**Figure 4:**
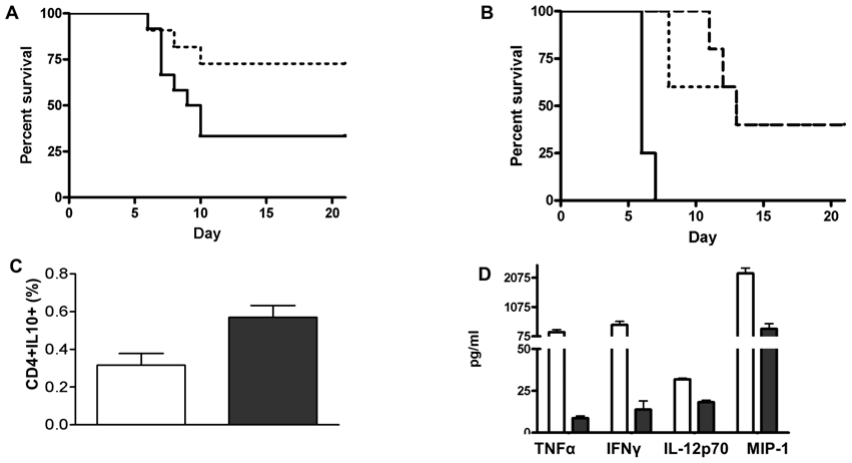
Outcomes of WNV infection by mosquito bite of rD7-vaccinated mice. A. Survival curves comparing WNV-infected rD7-vaccinated mice (solid line, n = 13) with mock-vaccinated mice (dotted line, n = 12). Results are combined from four experiments (p = 0.0347). B. Survival curves after passive serum transfer and infection with WNV. Mice were injected IP with pooled sera from rD7-vaccinated mice (solid line, n = 4), non-vaccinated mice (dotted line, n = 5), or mice repeatedly bitten by *Cx. tarsalis* (dashed line, n = 5) and 30 h later infected with WNV *via* bites of infected *Cx. tarsalis.* Mice passively immunized with rD7-vaccinee serum exhibited significantly higher mortality rates than either control group (p = 0.002). Mortality rates of mice passively immunized with mosquito-exposed mouse serum were not different from those that received untreated mouse serum. C. Proportion of total splenocytes from rD7- and mock-vaccinated mice that were CD4+ T-lymphocytes staining positive for intracellular IL-10 two days post-WNV infection; rD7-vaccinated animals (filled bar) had significantly higher IL-10 levels than mock-vaccinated animals (p<0.05, n = 3 per group). D. CBA assay of medium from WNV peptide-stimulated splenocytes that were collected at 2 days post-WNV infection; all cytokine levels from rD7-immunized mice (filled bars) were significantly lower than those from mock-immunized mice (open bars) (p<0.05, n = 3 per group).

To assess the protective effect of rD7 vaccination on subsequent WNV infection, we challenged vaccinated mice with WNV *via* bites of infected *Cx. tarsalis* at 10 days after the final immunization. Infected animals were monitored twice daily for 21 days for morbidity and mortality and were euthanized upon observation of severe neurologic signs. Surprisingly, we observed significantly reduced survival rates in rD7-vaccinated mice (33.3%) as compared to mock-vaccinated mice (72.7%, p = 0.03, **Figure 4A**). Moreover, we noted that mice vaccinated with rD7 developed more severe neurologic signs, including hunched posture, paralysis, whirling, and repetitive behaviors, than mock-vaccinated animals (data not shown). Viral load was determined by qRT-PCR in the brains and blood of the infected mice at 2 and 4 days post infection and, although the amount of WNV RNA was greater in all tissues of rD7-vaccinated mice, no statistical difference was noted between vaccinated and mock-vaccinated mice (**Figure S1**). These results suggest that vaccination of mice with rD7 protein enhances susceptibility to severe WNV disease, but does not dramatically increase viral replication.

To determine if the increased pathogenicity and mortality observed in rD7-vaccinated mice were due to humoral or cellular responses induced by vaccination, we injected naïve mice intraperitoneally with pooled sera from rD7-immunized mice one day before infection with WNV by bite of infected *Cx. tarsalis* mosquitoes. Mice injected with sera of mice that had been repeatedly exposed to *Cx. tarsalis* over the course of one year (naturally-exposed) or sera of non-vaccinated mice served as controls. Infected mice were monitored twice daily for 21 days. All mice with adoptively-transferred rD7-vaccinee sera succumbed to WNV infection within the first week after infection (0% survival). Mice receiving the naturally-exposed or untreated, non-exposed mice sera exhibited similar, higher survival rates (40%, p = 0.002, **Figure 4B**).

To study T cell responses, we harvested splenocytes from rD7-vaccinated, mock-vaccinated and non-immunized control mice at days 2 and 4 post-mosquito-delivered WNV infection and stimulated them with WNV specific peptides [27], [28], then stained with antibodies specific for IL-10, IL-4, IFNγ, TNFα, or IL-12. At day 2 post-infection, we observed an 84% higher proportion of IL-10-producing splenic CD4^+^ T cells from rD7-immunized mice compared to splenocytes of mock-immunized mice (p = 0.016, **Figure 4C**). We did not detect significant differences between groups in other cytokine staining at day 2 or day 4 post infection (data not shown). At 2 dpi, release into medium of Th1-type cytokines (TNFα, IFNγ, IL-12 and MIP-1) by CD4+ T cells from rD7-vaccinated mice was significantly reduced compared to mock-vaccinated mice (**Figure 4D**). At day 4 post-infection, we observed an increase in the proportion of splenocytes from rD7-vaccinated mice stimulated with the MHC class I/CD 8+ peptide that were producing IL-4 (p = 0.01; data not shown). There were no significant differences between groups at 2 dpi or in other cytokines at 4 dpi in CD8+ peptide-stimulated cells. Overall, our results suggest that upon mosquito-transmitted WNV infection, rD7 vaccinated mice have enhanced host susceptibility to severe disease, possibly due to their anti-rD7 antibody response, decreased Th1-type cytokine production and enhanced proportion of IL-10 producing CD4+ T cells.

## Discussion

Natural feeding of *Culex* spp. and *Aedes* spp. mosquitoes on flavivirus-susceptible C3H/HeJ mice has been shown to down-regulate Th1-type cytokines and up-regulate Th2 cytokines [12], which are not favorable for an effective immune defense against infection by arthropod-borne viruses [13], [14], [15], [16]. Our prior studies showed that mice vaccinated with *Cx. tarsalis* salivary gland extract and subsequently challenged with WNV by mosquito bite had decreased viral titers in the brain early in infection (Machain-Williams et al., submitted). These results suggested that development of and immunization with a MSP vaccine could potentially neutralize saliva-induced immunomodulation and protect the host from mosquito-transmitted WNV infection. D7 family proteins are specifically expressed in the salivary glands of adult female hematophagous diptera [29], are immunogenic [18] and are among the most abundant proteins in the saliva of female mosquitoes [30], [31], comprising between 5 and 20% of total salivary protein [32]. These characteristics led us to choose a D7 family protein as our vaccine candidate. As we began our study, *Cx. tarsalis* D7 salivary proteins had neither been isolated nor characterized. We identified 36 kDa and 40 kDa proteins by mass spectrometry that have significant similarity to two *Cx. pipiens* D7 salivary proteins. We chose to focus on the 36 kDa protein, which had 85% amino acid sequence identity to a *Cx. pipiens* D7 salivary protein. The cDNA encoding the 36 kDa protein was cloned, sequenced and used to express recombinant protein by an alphavirus transducing system in cultured *Aedes albopictus* cells. This system has the advantage of abundant expression in mosquito cells, which should yield authentic native protein.

Vaccinated mice exhibited slightly increased expression of IFN-γ and significantly reduced expression of IL-10 shortly after uninfected mosquito bite. These results, along with our observation of more inflammatory cell infiltration at the bite site upon non-infected mosquito feeding suggested that rD7 protein immunization might favorably alter the immune response to WNV infection delivered by mosquito bite.

To our surprise, rD7 vaccinated mice that were subsequently bitten by WNV-infected mosquitoes exhibited higher mortality rates than mock- immunized mice. Passive immunization of mice with sera from rD7-vaccinated mice also resulted in increased mortality due to mosquito-delivered WNV infection compared to mice that received serum from non-immunized mice or were passively immunized with sera of naturally mosquito-exposed mice. These results indicate that vaccine-induced antibodies specific to D7 protein or other serum components play a predominant role in viral pathogenesis in immunized mice. Schneider *et al* [17] demonstrated that prior exposure to *Aedes aegypti* mosquito bites or passive transfer of serum from mosquito-exposed mice increased the proportion of animals that succumbed to *Ae. aegypti*-transmitted WNV infection. One possible reason for these observations is that high levels of circulating antibodies to MSPs could impede mosquito blood-feeding by neutralization of vasodilator and platelet inhibitor functions. This could lead to increased probing time and thus more virus-containing saliva being deposited, resulting in an increased initial infectious virus dose. Differences in effects of passive transfer of serum from mosquito-exposed mice on outcome of subsequent WNV infection seen in our study could be due to differences in doses, routes and duration of exposure to MSP as well as to the mosquito genera and species. In our study, although rD7-immunized mice had an anti-D7 IgG response equivalent to that of mice repeatedly exposed to mosquito bites, they produced only IgG1 and not IgG2a anti-D7 antibodies, as did naturally-exposed mice. IgG1 and IgG2a subtypes are normally made during Th2- and Th1-type immune responses, respectively. Th1-type CD4+ T-cells have been shown to produce IL-12p70 and IFNγ, which promote class switching of antibodies from IgM to IgG2a. In contrast, Th2-type T-cells characteristically produce IL-4 that directly inhibits the Th1 response and induces class switching to IgG1. A Th2-dominated response is usually seen after mosquito bite; however, administration of a MSP vaccine with complete Freund's adjuvant (CFA) was expected to result in a Th1 type response [33] and generation of IgG2a antibodies. The D7 protein may drive the development of IgG1 subtype antibodies regardless of the use of CFA, as has been reported before [34]. In fact, we observed a larger proportion of IL-10-producing WNV-specific CD4^+^ T-cells from rD7-vaccinated infected mice and significant decreases in pro-inflammatory cytokine production by CD4+ T cells. Pro-inflammatory cytokines are essential to effectively respond to WNV infection [13], and IL-10 is known to be involved in WNV pathogenesis [16], [17].

Histological examination at the site of an uninfected *Cx. tarsalis* bite showed that rD7-vaccinated mice had moderately severe inflammation and PMN and mononuclear cell infiltration. Monocytes and dendritic cells are susceptible to WNV infection [35], [36]. A local increase in susceptible cells at the site of WNV injection could make more cells available for immediate infection and migration to regional lymph nodes and thereby result in more virus spread in the periphery. Overall, immunization of mice with rD7 protein induced exclusively IgG1 anti-D7 responses, more inflammatory cell infiltration at the site of mosquito feeding and did not result in a shift to Th1-type cytokine production after WNV infection, which together could contribute to enhanced host susceptibility to virus infection and disease. This response failed to favorably alter the immunomodulatory effect of MSP on WNV infection.

Our prior studies showed that mice vaccinated with *Cx. tarsalis* salivary gland extract and subsequently challenged with WNV initially had decreased viral titers in the brain (Machain-Williams *et al*., submitted), suggesting that a MSP vaccine was potentially protective against WNV infection. Nevertheless, our studies presented here showed that immunization of mice with the 36 kDa rD7 protein resulted in enhanced mortality upon WNV infection. It appears that the 36 kDa D7 protein is not the right candidate for a MSP vaccine and its selection on the bases of abundance and immunogenicity did not predict efficacy. Future studies should focus on other MSPs. These findings illustrate the complex nature of the immune response to salivary proteins and effects this may have on arbovirus transmission and infection and provide important information to direct future studies in the field of immunomodulatory effects of mosquito saliva.

## Supporting Information

Figure S1 **WNV E-protein RNA levels.** WNV RNA was measured via quantitative PCR at 2 and 4 dpi in blood and brains of mock- and rD7-vaccinated mice. Although the amount of WNV RNA was greater in all tissues of rD7-vaccinated mice, the differences were not statistically significant. (n = 3 per group).
(TIF)
