# Supplementary figures and images for: Immunization of Mice with Recombinant Mosquito Salivary Protein D7 Enhances Mortality from Subsequent West Nile Virus Infection via Mosquito Bite
Source: PLoS Negl Trop Dis. 2012 Dec 6;6(12):e1935. doi: 10.1371/journal.pntd.0001935 (PMC3516580; doi:10.1371/journal.pntd.0001935)

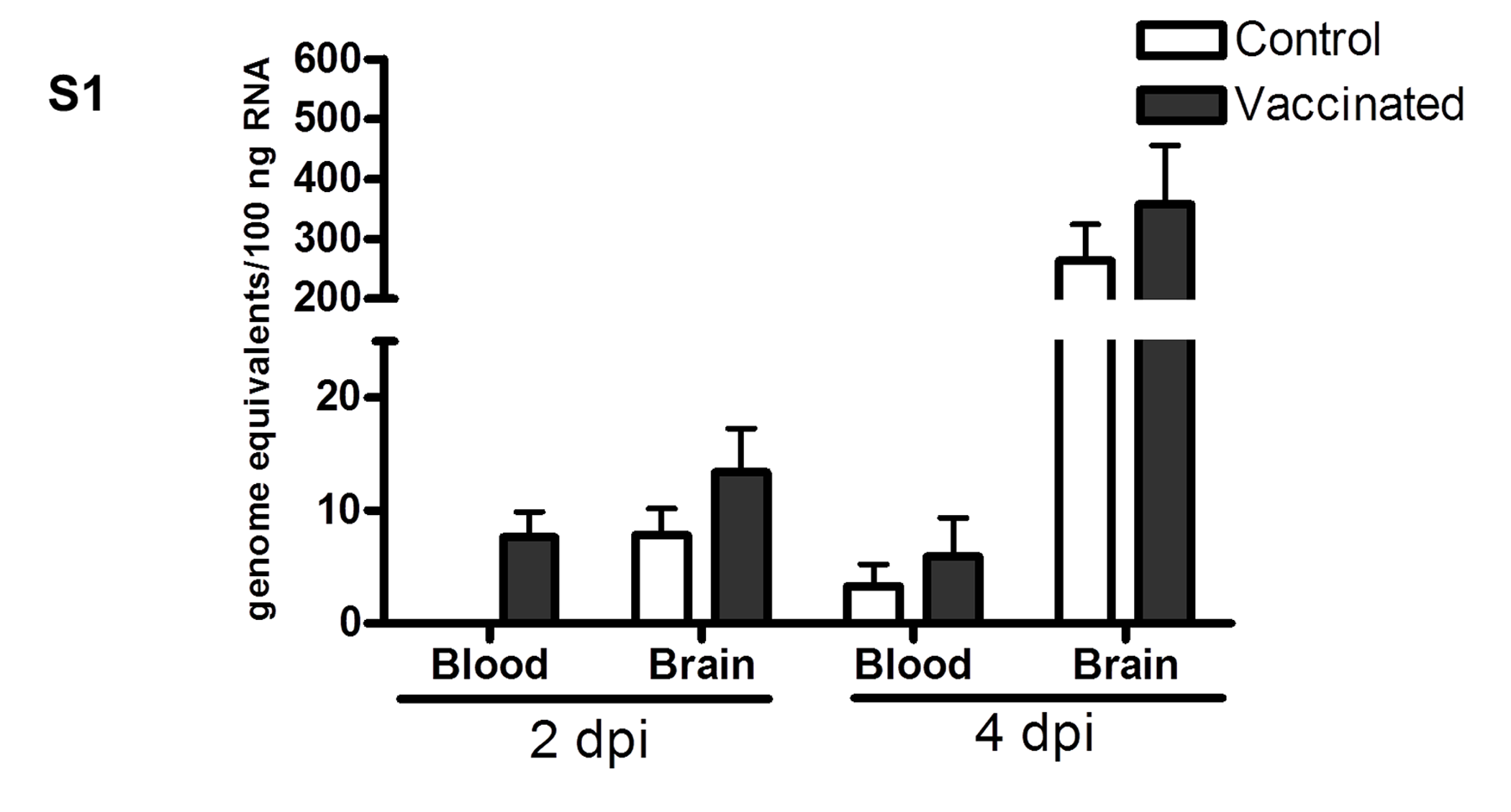

Supplement: Figure S1 — WNV E-protein RNA levels. WNV RNA was measured via quantitative PCR at 2 and 4 dpi in blood and brains of mock- and rD7-vaccinated mice. Although the amount of WNV RNA was greater in all tissues of rD7-vaccinated mice, the differences were not statistically significant. (n = 3 per group). (TIF) [file pntd.0001935.s001.tif]
